# Copy Number Variation and Expression Analysis Reveals a Nonorthologous *Pinta* Gene Family Member Involved in Butterfly Vision

**DOI:** 10.1093/gbe/evx230

**Published:** 2017-11-09

**Authors:** Aide Macias-Muñoz, Kyle J McCulloch, Adriana D Briscoe

**Affiliations:** Department of Ecology and Evolutionary Biology, University of California, Irvine; FAS Center for Systems Biology, Harvard University

**Keywords:** phototransduction, chromophore, retinol-binding proteins, tandem duplication, copy number variations

## Abstract

Vertebrate (cellular retinaldehyde-binding protein) and *Drosophila* (prolonged depolarization afterpotential is not apparent [PINTA]) proteins with a CRAL-TRIO domain transport retinal-based chromophores that bind to opsin proteins and are necessary for phototransduction. The CRAL-TRIO domain gene family is composed of genes that encode proteins with a common N-terminal structural domain. Although there is an expansion of this gene family in Lepidoptera, there is no lepidopteran ortholog of *pinta*. Further, the function of these genes in lepidopterans has not yet been established. Here, we explored the molecular evolution and expression of CRAL-TRIO domain genes in the butterfly *Heliconius melpomene* in order to identify a member of this gene family as a candidate chromophore transporter. We generated and searched a four tissue transcriptome and searched a reference genome for CRAL-TRIO domain genes. We expanded an insect CRAL-TRIO domain gene phylogeny to include *H. melpomene* and used 18 genomes from 4 subspecies to assess copy number variation. A transcriptome-wide differential expression analysis comparing four tissue types identified a CRAL-TRIO domain gene, *Hme CTD31*, upregulated in heads suggesting a potential role in vision for this CRAL-TRIO domain gene. RT-PCR and immunohistochemistry confirmed that *Hme CTD31* and its protein product are expressed in the retina, specifically in primary and secondary pigment cells and in tracheal cells. Sequencing of eye protein extracts that fluoresce in the ultraviolet identified Hme CTD31 as a possible chromophore binding protein. Although we found several recent duplications and numerous copy number variants in CRAL-TRIO domain genes, we identified a single copy *pinta* paralog that likely binds the chromophore in butterflies.

## Introduction

Phenotypic differences between organisms may be driven by small nucleotide changes in protein coding or regulatory regions, or by whole gene or genome duplications (Stern 2000; Hersh and Carroll 2005; Demuth et al. 2006). Gene duplications in particular are hypothesized to be an important mechanism for evolutionary change because these events give rise to new material for novelties and may facilitate the emergence of new genes (Ohno 1970; Long et al. 2003). Often, gene duplications result in pseudogenization. However, there are at least two mechanisms by which duplicated genes can remain functional regardless of redundancy: 1) in neofunctionalization, a duplicated gene develops a new function different from the ancestral gene and 2) in subfunctionalization the two paralogs each have part of the function of an ancestral gene (Lynch and Conery 2000; Long et al. 2003; Zhang 2003). Gene duplications and rearrangements have resulted in large gene families. Genes are classified as part of a gene family when they share common sequence motifs and sometimes may have related general functions (Henikoff et al. 1997).

Lineage-specific gene family expansions are hypothesized to be a mechanism by which eukaryotic species can adapt and diversify (Lespinet et al. 2002). In support of this, studies in mammals suggest that changes to the size of large gene families are likely arising through lineage specific gene loss or gain rather than by changes in gene number at branch sites (Demuth et al. 2006). Gene families that are subject to expansions or reductions have a wide variety of functions, including immunity and sensory perception (Cooper et al. 2007; Dopman and Hartl 2007; Conrad et al. 2010). Chemosensory genes in particular have been widely studied in a number of species and have been found to vary in copy number between and within species (Nei et al. 2008; Nozawa and Nei 2008). Copy number variation (CNV) is a DNA segment 1 kb or longer whose copy number varies between individuals, as a result of recent gene duplications or deletions (Stranger et al. 2007). Insects have been studied for CNV by focusing on gene families with lineage-specific duplications; these genes are candidates for CNVs (Zhang 2003). As an example, the butterfly *Heliconius melpomene* and the pea aphid *Acyrthosiphon pisum* both have lineage-specific gene expansions and CNV of olfactory and gustatory receptors correlated with host plant specialization (Briscoe et al. 2013; Duvaux et al. 2015).

The CRAL-TRIO domain gene family is another family that is evolving by lineage-specific duplication in insects and has undergone an expansion in Lepidoptera (moths and butterflies; Smith and Briscoe 2015). Lepidoptera thus have almost twice as many CRAL-TRIO domain genes relative to other insects (Smith and Briscoe 2015). The lineage-specific duplications of this gene family make it a candidate to study for CNV (Zhang 2003). Furthermore, the specific functions of the members of this family remain unknown, with one or two exceptions. The CRAL-TRIO domain is an N-terminal structural region, ∼170 amino acids long, common to several proteins that bind and transport tocopherols (Panagabko et al. 2003; Sigrist et al. 2012). The CRAL-TRIO domain gene family includes a cellular retinaldehyde-binding protein (CRALBP) which is essential to vertebrate vision due to its function in chromophore transport (Wu et al. 2006). The visual pigment chromophore is derived from vitamin-A. In photoreceptor cells, opsin proteins bind a chromophore molecule (in humans 11-*cis*-retinal and in butterflies 11-*cis*-3-hydroxyretinal) to form rhodopsin. Rhodopsin initiates the phototransduction cascade when photon absorption changes the chromophore configuration from 11-*cis* to all-*trans* (von Lintig et al. 2010). In humans, mutations to CRAL-TRIO domain genes result in a variety of retinal and neurological diseases (Maw et al. 1997; Bomar 2003; Min et al. 2003). Mutations in RLBP1, the gene encoding CRALBP in humans, results in retinitis pigmentosa (Maw et al. 1997) and mutations in a gene encoding αTTP results in ataxia with vitamin E deficiency (AVED) (Min et al. 2003). Moreover, mutations in human *Atcay*, a CRAL-TRIO domain containing gene, are associated with Cayman ataxia, and a mouse homolog of *atcay* causes ataxia and dystonia in jittery mice (Bomar 2003).

In insect genomes, CRAL-TRIO domain genes are numerous, however, their function remains largely unexplored except for *prolonged depolarization afterpotential is not apparent* (*pinta*). PINTA in *Drosophila* is a CRAL-TRIO domain protein belonging to the SEC14 superfamily that, similar to CRALBP, shuttles the chromophore from retinal pigment cells to photoreceptor cells (Wang and Montell 2005). PINTA protein is required for the biosynthesis of rhodopsin. *Drosophila* with mutated *pinta* genes have low expression of Rh1, the protein component of the light-sensitive rhodopsin found in R1-6 photoreceptor cells (Wang and Montell 2005). Similarly, another member of the SEC14 superfamily squid RALBP functions in retinal binding in cephalopods (Ozaki et al. 1994; Speiser et al. 2014). Although there is an expansion of CRAL-TRIO domain genes in Lepidoptera, no *pinta* ortholog has been found in this group. The functions of CRALBP and PINTA suggest that a distinct CRAL-TRIO domain protein might be serving an essential role in lepidopteran visual systems by transporting the chromophore.

Presently, most of our knowledge about photoreceptor determination, phototransduction, and chromophore transport comes from studies in *Drosophila*. However, a recent analysis of 80 vision genes in the *Manduca sexta* genome (Kanost et al. 2016) found that at least four gene families involved in photoreceptor differentiation pathways have undergone lepidopteran-specific gene duplications including *corkscrew*, *embryonic lethal/abnormal vision*, rhabdomeric opsins, and genes encoding CRAL-TRIO domain containing proteins. Since CRAL-TRIO domain genes have undergone an expansion in Lepidoptera and their functions in other organisms suggest a role in vision, it is worth exploring the function of these genes in a butterfly species. *Heliconius melpomene* provides a good system in which to investigate the evolution of CRAL-TRIO domain genes due to the availability of a reference genome and a growing collection of resequenced genomes (Martin et al. 2013; Davey et al. 2016). In addition, we have generated RNA-Seq data from *Heliconius melpomene* tissues with which to investigate the expression of the CRAL-TRIO domain genes.

Here, we aim to 1) characterize the molecular evolution of the CRAL-TRIO domain gene family and to 2) identify a candidate gene for chromophore transport in butterflies. We used RNA-Seq data from *H. melpomene* head, antennae, legs, and mouth parts to make a de novo transcriptome assembly from which to identify CRAL-TRIO domain gene transcripts. We also investigated the reference genome to search for any CRAL-TRIO domain genes that may be found in the genome but not expressed in the tissues we sampled. We found support for the expansion of the CRAL-TRIO domain gene family in butterflies by identifying 43 CRAL-TRIO domain genes in the *H. melpomene* genome comparable with the 42 found in *Manduca sexta* (Smith and Briscoe 2015). We also investigated 18 resequenced *H. melpomene* genomes (Martin et al. 2013) for structural variation (specifically CNV) and found that 32 of the 43 genes in the reference genome had either a large duplication or deletion in at least one of the resequenced genomes. Further, to identify a CRAL-TRIO domain gene functioning in vision, we did a differential expression analysis between tissue types and found one CRAL-TRIO domain gene (*Hme CTD31*) that is upregulated in head tissue. RT-PCR and immunohistochemistry shows that *Hme CTD31* is expressed in the compound eye and not the brain, and Hme CTD31 is localized to the retinal pigment and trachea cells making it a candidate chromophore binding protein. We used mass spectrometry to sequence eye proteins associated with an ultraviolet fluorescing pigment and found a match for our CRAL-TRIO domain protein Hme CTD31. These various lines of evidence suggest that we have found a CRAL-TRIO domain gene that binds the chromophore in butterflies.

## Materials and Methods

### CRAL-TRIO Domain Gene Phylogeny

A phylogeny (Smith and Briscoe 2015) was expanded by adding homologs of the CRAL-TRIO domain gene family found in *H. melpomene*. Smith and Briscoe identified CRAL-TRIO domain genes from the genomes of *Manduca sexta*, *Danaus plexippus*, *Drosophila melanogaster*, *Anopheles gambiae*, *Apis mellifera*, *Tribolium castaneum*, and *Bombyx mori* (Smith and Briscoe 2015). To expand this repertoire, we used BLAST + (Basic Local Alignment Search Tool) (Camacho et al. 2009) to identify CRAL-TRIO domain gene homologs in a de novo transcriptome of *H. melpomene rosina*. These contig sequences were extracted and added to the alignment. Contig nucleotide sequences were translated and curated in MEGA by finding the correct reading frame from start to stop codon. Sequences with missing homologs were blasted against the *H. melpomene melpomene* reference genome v. 2 (Davey et al. 2016), from which additional sequences were recovered. Manual annotations of the genes not included in the transcriptome and not annotated in the reference genome were done by extracting the nucleotide sequence around the area where there was a BLAST hit to a CRAL-TRIO domain gene. The extracted nucleotides were annotated and translated in AUGUSTUS (Stanke and Morgenstern 2005) and aligned to a BLAST output of the genome to correct the sequence. 215 amino acid sequences were aligned using MUSCLE (Edgar 2004) with default settings, and this alignment was then modified manually. A Bayesian phylogenetic tree was made using MrBayes (Huelsenbeck and Ronquist 2001; Ronquist and Huelsenbeck 2003; Ronquist et al. 2011) with a BLOSUM62 (Henikoff and Henikoff 1992) model for 1,000,000 generations. The phylogeny was color coded using iTOL (Letunic and Bork 2016).

### Structural Variation

To detect copy number variation (CNV) in these genes, we aligned reads for 18 resequenced *H. melpomene* genomes generated by Martin et al. (Martin et al. 2013), European Nucleotide Archive: ERP002440. Read mapping to the reference genome for four subspecies (six *H. melpomene melpomene*, four *H. melpomene rosina*, four *H. melpomene amaryllis*, and four *H. melpomene aglaope*) was performed using bwa (Li and Durbin 2009), and samtools was used to index and sort the files (Li et al. 2009). Pindel was used to examine mapping results to detect structural variation (Ye et al. 2009). Pindel looks for read pairs for which one read maps uniquely to the genome while the other read is unmapped to determine the structural breakpoint and direction of unmapped reads (Ye et al. 2009).

### RNA Library Preparation

We extracted RNA from whole heads (excluding antennae and mouth parts) of three male and three female *H. melpomene* butterflies. We also extracted RNA from the head, antennae, legs, and mouth parts (lapial palps + proboscis) of one male and one female *H. melpomene* specimen to increase our biological replicates to *n* = 4. Butterflies were placed in −80 °C and stored until RNA extraction. RNA was extracted using TRIzol (Life Technologies, Grand Island, NY) and purified using a NucleoSpin RNA II kit (Macherey-Nagel, Bethlehem, PA). Purified RNA was quantified using a Qubit 2.0 Fluorometer (Life Technologies, Grand Island, NY) and quality checked using an Agilent Bioanalyzer 2100 (Agilent Technologies, Santa Clara, CA). A TruSeq RNA Sample Preparation Kit v2 (Illumina, San Diego, CA) was used to prepare sequencing libraries. Libraries with distinct adapter sequences were quantified, quality checked, normalized, and pooled according to their concentrations. Pooled libraries were run on a 2% agarose gel. A Geneclean III kit (MP Biomedical, Santa Ana, CA) was used to recover DNA from the gel (∼240–600 bp), and Agencourt AMPure XP (Beckman Coulter, Brea, CA) beads were used for a second purification. Sequencing was conducted at the UCI Genomics High-Throughput Facility using a HiSeq 2500 (Illumina, San Diego, CA), paired end 100-cycle sequence run.

### Assembly and Read-Mapping

RNA-Sequencing data were obtained for three *H. melpomene* males and three female antennae, legs, and mouth parts from a previous RNA-Seq study (ArrayExpress: E-MTAB-1500) (Briscoe et al. 2013). We created eight new head libraries from four males and four females. In addition, we made a new antennae, legs, and mouth parts library for one *H. melpomene* male and one female. The raw sequencing data for the 14 new libraries were deposited in ArrayExpress archive under E-MTAB-6249 and E-MTAB-6342. All libraries were parsed using custom perl and python scripts. A de novo transcriptome assembly was constructed using Trinity (Grabherr et al. 2011; Haas et al. 2013) by including one library per tissue type (head, legs, antennae, mouthparts) for one male and one female, eight libraries total. We made a de novo assembly because the CRAL-TRIO domain genes were not all annotated in the genome and a transcriptome recovered more sequences that were complete. The reference transcriptome was deposited in Dryad under doi: 10.5061/dryad.857n9. Each sequenced library was then mapped back to the reference assembly using RSEM (Li and Dewey 2011) from which we extracted raw read count data, FPKM (Fragments Per Kilobase of exon per Million fragments mapped), and TPM (Transcripts Per Kilobase Million). FPKM was further normalized using NOISeq (Tarazona et al. 2011).

Since some of the CRAL-TRIO domain genes were not recovered in the transcriptome, we manually annotated these genes and read mapped each library as described earlier to the nucleotide sequences of the 43 genes. TPM expression was scaled to the values of whole-transcriptome analysis. We then used two-way ANOVAs to test if these genes varied by sex, tissue type, or sex and tissue type interaction.

### edgeR

We performed differential gene expression analysis for all Trinity assembled contigs using edgeR (Robinson and Smyth 2007, 2008; Robinson et al. 2010; Mccarthy et al. 2012). To analyze genes differentially expressed by tissue type, we did pairwise comparisons of head versus antennae, head versus legs, and head versus mouth parts using a generalized linear model with terms for tissue, sex, the interaction of sex and tissue and included a term to correct for batch effects of sequencing on different lanes (∼batch + tissue + sex + sex×tissue). Each analysis included filtering to remove contigs expressed at <1 count per million (CPM) for at least four groups, and between sample normalization using a trimmed mean of the log expression ratios (TMM) (Robinson and Oshlack 2010). Contigs were considered significantly differentially expressed when the false discovery rate (FDR) was <0.05 and the log fold change (logFC) was >1. We did FDR corrections using the qvalue package and using a Bonferroni correction (Storey and Tibshirani 2003; Dabney and Storey 2013).

### RT-PCR

To localize where in the head the candidate gene was expressed, we performed reverse transcription polymerase chain reaction (RT-PCR) using RNA from a single individual male and female *H. melpomene* antennae, retina, and brain tissue. Animals were sacrificed a day after eclosion by squeezing the thorax. Heads were dissected in petri dishes in Ringer’s solution, the retina and brain tissue were transferred to 1.7-ml microtubules on ice. Total RNA was extracted from these tissues using TRIzol (Life Technologies, Grand Island, NY) and quantified using a Qubit 2.0 Fluorometer (Life Technologies, Grand Island, NY). RNA was treated with DNAse I (Fisher Scientific, Pittsburgh, PA). Primers were designed using Primer3 (supplementary table S1, Supplementary Material online) (Koressaar and Remm 2007; Untergasser et al. 2012). Each 25 μl reaction had 2.5 μl Choice PCR buffer (Denville Scientific, South Plainfield, NJ), 2.5 μl dNTPs (2 mM), 0.5 μl Choice-Taq Blue (Denville Scientific, South Plainfield, NJ), 0.5 μl (1:20 diluted) SuperScript II Reverse Transcriptase (Life Technologies, Grand Island, NY), 0.5 μl forward primer (10 μM), 0.5 μl reverse primer (10 μM), 18 μl H_2_O, and 1 μl RNA (12 μg/ml). The PCR reaction consisted of 42 °C for 30 min, 20 cycles of (95 °C for 30 s, 55 °C for 30 s, and 68 °C for 55 s), 68 °C for 7 min, 4 °C hold. We visualized amplification by running the PCR products on a 2% agarose gel.

### Immunohistochemistry

An antibody against the peptide N-CLRPGKPTNYDELFGID-C of the *Heliconius melpomene* CTD31 was generated in chicken and immunoaffinity purified (New England Peptide, Gardner, MA). We also used a rabbit antibody against the nymphalid *Limenitis astyanax* LWRh opsin sequence (Frentiu et al. 2007) to label LWRh expressing cells in *H. melpomene* (McCulloch et al. 2016)*.* Eyes were fixed, sucrose protected, cryosectioned, and immunolabeling was performed as described in McCulloch et al. (2016). Slides were placed in 100% ice-cold acetone for 5 min, then washed 3 × 10 min in 0.1 M Phosphate-buffered saline (PBS). Slides were then placed in 0.5% sodium dodecyl sulfate in 0.1 M PBS for 5 min. Each slide was blocked for 1 h at room temperature using 8% (v/v) normal goat serum, and 0.3% Triton X-100 in 0.1 M PBS. Slides were incubated with 1:75 chicken anti-CTD31 and 1:15 rabbit anti-LWRh antibodies in blocking solution overnight at 4 °C. Slides were washed 3 × 10 min in 0.1 M PBS and then incubated with 1:1,000 goat antichicken Alexafluor 488 and 1:500 goat antirabbit Alexafluor 555 secondary antibodies in blocking solution for 2 h at room temperature in the dark. Slides were washed once more 3 × 10 min in 0.1 M PBS in the dark. Slides were stored for imaging by coverslipping with Aqua Poly/Mount (Polysciences, Inc. Cat. # 18606). Image stacks were taken using a Zeiss LSM700 Confocal Microscope under 20× objective at the UC Irvine Optical Biology Core Facility. Maximum intensity projections and two-channel composites were generated using Fiji. Brightfield images were taken using untreated sections and were viewed with epifluorescence microscopy using a Zeiss Axioskop 2 under a 20× lens. Images were taken using a Canon PowerShot S5 and associated Canon software. Contrast and brightness were adjusted for clarity using Adobe Photoshop and Fiji.

### Western Blot and Mass Spectrometry

Butterfly heads were removed and immediately placed at −80°C until they were shipped together with an aliquot of anti-CTD31 antibody to Zyagen (San Diego, CA) overnight on dry ice. Immunoblotting was performed by Zyagen. Proteins were extracted by mechanical homogenization in protein lysis buffer and estimated protein concentration using a BCA kit. Total protein was fractionated through two large gels (SDS–PAGE) at different concentrations (20, 40, 60, and 80 μg each gel). Protein from the two gels was then transferred to Polyvinylidene fluoride (PVDF) membranes. The membrane of the first gel was blocked with 5% milk in TBST for 2 h, then incubated with primary antibody anti-CTD31 at a concentration of 1:100 at 4 °C overnight. Membrane was washed 3 times in TBST then incubated with secondary antibody (antichicken-peroxidase antibody) from Jackson ImmunoResearch (West Grove, PA) at a concentration of 1:5,000 for 1 h. After several washes, membrane was incubated for 5 min with chemiluminescence substrate. Two major protein bands were observed ∼35 kDa.

To visualize which protein may be interacting with the chromophore, eight aliquots (50 μg each) of butterfly head proteins were fractionated through a large native gel by Zyagen (San Diego, CA). One lane was cut and visualized on UV light to locate bands that fluoresce. Gel pieces containing three protein bands were collected in 15-ml tubes and were shipped to UC Irvine. The samples were immediately transferred to a Proteomics & Mass Spectrometry Facility in the school of Biological Sciences (Irvine, CA) for nano LC-MS/MS mass spectrometry using an LTQ Velos Pro mass analyzer (Thermo-Fisher). The resulting peaklists were compared against our translated transcriptome along with a database of common contaminants using Mascot 2.5 to score (Matrix Science, Boston, Massachusetts). Mascot scores are the probability that the ion score of the experimental data match the ion scores of the database sequence; protein scores <67 are significant (*P* < 0.05).

## Results and Discussion

### Phylogeny and Chromosomal Location

**Fig. 1.**
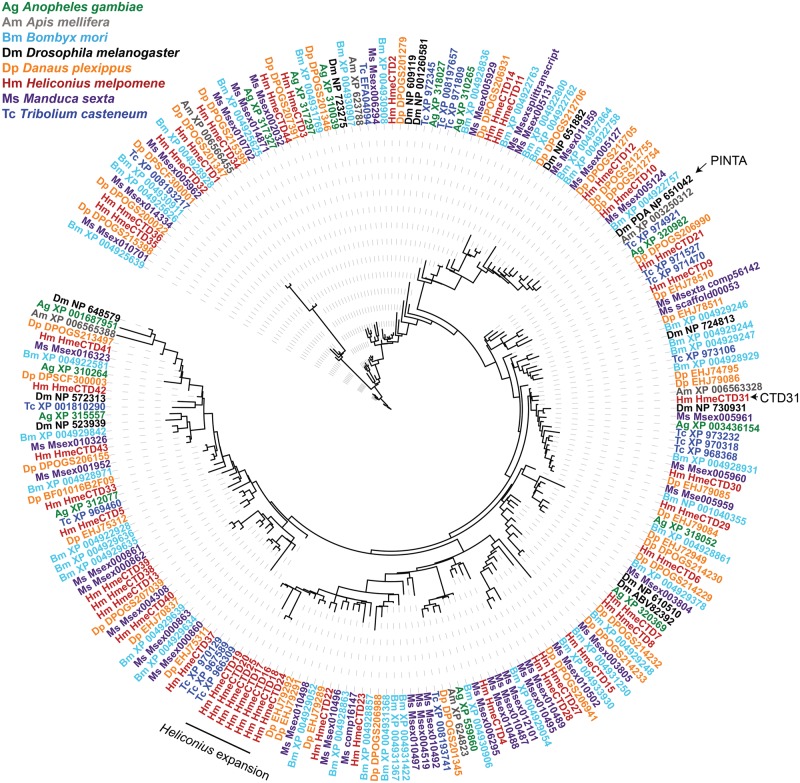
**—Bayesian phylogeny of insect CRAL-TRIO domain proteins**. Phylogeny includes sequences from *Anopheles gambiae* (green), *Apis mellifera* (gray), *Bombyx mori* (light blue), *Drosophila melanogaster* (black), *Danaus plexippus* (orange), *Heliconius melpomene* (red), *Manduca sexta* (purple), and *Tribolium casteneum* (dark blue). The Bayesian tree was found using MrBayes with a BLOSUM62 model of amino acid substitution. The *Heliconius* expansion as well as *Drosophila pinta* and *Heliconius Hme CTD31* are indicated on the phylogeny with black lines and arrows.

**Fig. 2.**
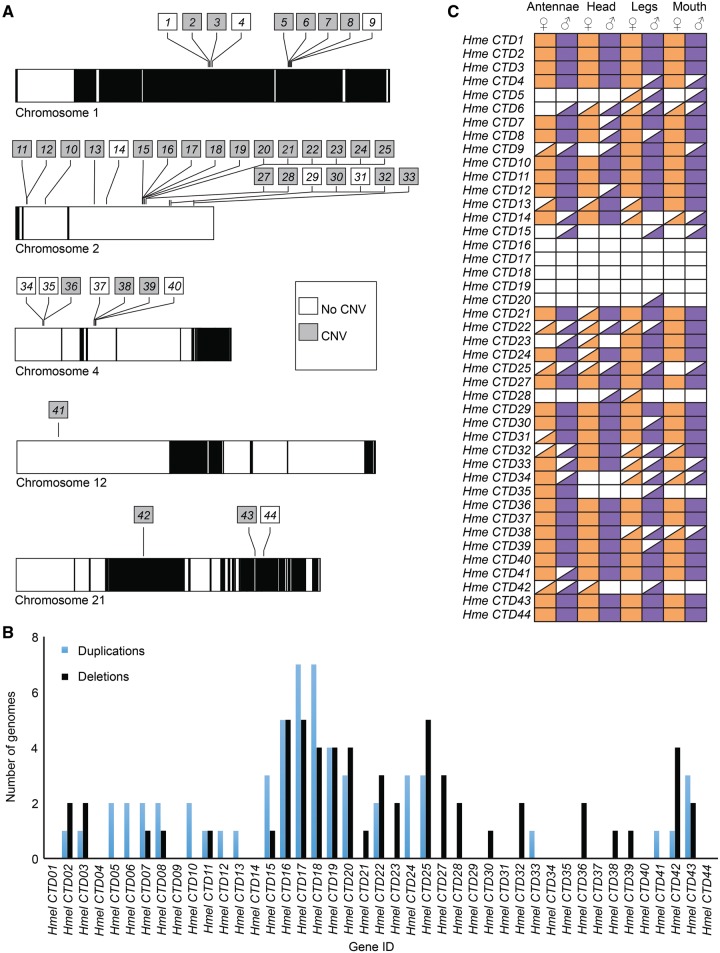
**—CRAL-TRIO domain gene location, copy number variation, and mRNA presence patterns**. (*A*) CRAL-TRIO domain genes are located on five chromosomes, many in tandem. Alternating black and white chromosomal regions represent scaffolds. Shaded squares represent genes with copy number variation, duplicated and/or deleted in at least 1 of 18 resequenced genomes. (*B*) Number of genomes in which CRAL-TRIO domain genes are deleted (black) or duplicated (blue) in 18 *Heliconius melpomene* resequenced genomes. (*C*) mRNA presence patterns of CRAL-TRIO domain genes in *H. melpomene* male and female antennae, head, legs, and mouth parts. Filled square represents complete presence (>1 TPM for all replicates), half-filled square represents partial presence (>1 TPM for at least one replicate but not all four), and no fill represents lack of transcript mRNA.

We identified a total of 43 CRAL-TRIO domain genes (*Hme CTD*) in the *H*. *melpomene* reference genome and 28 of them were recovered in a de novo assembly (supplementary table S2, Supplementary Material online). We found *H. melpomene* orthologs of most previously identified insect CRAL-TRIO domain genes (Smith and Briscoe 2015). We also discovered a recent duplication (*Hme CTD38* and *CTD39*) since *Heliconius* shared a common ancestor with *Danaus plexippus*, and an expansion of CRAL-TRIO domain genes (*Hme CTD16-20* and *Hme CTD24-25*; fig. 1). We refer to recent paralogs found in the reference genome as recent duplications; we refer to multiple duplications as an expansion, and genes with CNV are those that are duplicated or deleted in resequenced genomes compared with the reference. We named the *H. melpomene* CRAL-TRIO domain genes according to their location on scaffolds and since many genes are on similar scaffolds, we decided to map these genes on to chromosomes (fig. 2*A*). We found that all 43 genes were located on a total of 5 chromosomes and 23 of the genes were on a single chromosome, chromosome 2 (fig. 2*A*). Only one gene in this family (*Hme CTD44*) is intronless and likely arose through retrotransposition (Zhang 2003).

New genes also arise by tandem duplication which themselves arise by unequal crossing over resulting in new gene copies adjacent to each other or by segmental duplications which can be dispersed throughout the genome and experience few recombination events (Jelesko et al. 1999; Baumgarten et al. 2003; Zhang 2003; Cannon et al. 2004). Most of the CRAL-TRIO domain genes were located in tandem suggesting that this gene family is the result of early segmental duplications and recent tandem duplications or early and recent tandem duplications with rearrangements in *H. melpomene* (fig. 2*A*). Moreover, areas of gene duplication can be hotspots for chromosomal rearrangement and might be enriched for copy number variation (CNV) (Sharp et al. 2005). In *D. melanogaster*, tandem duplications are significantly enriched near areas with CNVs (Dopman and Hartl 2007). The physical locations of CRAL-TRIO domain genes display arrays of tandem duplications making this gene family a good candidate for studying CNV (Redon et al. 2006).

### Copy Number Variation

We used Pindel (Ye et al. 2009) to look for duplications and deletions 1 kb or larger (Stranger et al. 2007) of these CRAL-TRIO domain genes in resequenced genomes of four *H. melpomene* subspecies, *H. melpomene melpomene*, *H. melpomene rosina*, *H. melpomene amaryllis*, and *H. melpomene aglaope* (Martin et al. 2013). The average size of these genes including introns was 3,648 bp, coding sequences being ∼304 amino acids long. Nine genes (*Hme CTD1-9*) were located on chromosome 1; *Hme CTD2*, *3* and *5-8*, had potential CNV in at least 1 of the 18 sampled genomes (fig. 2*A*). *Hme CTD2-3* were duplicated in one *H. melpomene aglaope* individual and were deleted in two genomes (*H. m. melpomene* and *H. m. aglaope*; fig. 2*B* andsupplementary tables S3 and S4, Supplementary Material online). *Hme CTD5*-*8* were duplicated in two genomes (*H. m. melpomene* and *H. m. aglaope*; fig. 2*B* andsupplementary table S3, Supplementary Material online), but *Hme CTD7-8* were deleted in one *H. m. amaryllis* genome (supplementary table S4, Supplementary Material online).

Twenty-three genes (*Hme CTD10-33*) were located on chromosome 2 with more complex patterns of CNV. *Hme CTD10-13*, *15-20*, *22*, *24-25*, and *33* were potentially duplicated in one or more resequenced genome (fig. 2*B*). Of these duplicates, *Hme CTD11-13* and *33* were duplicated in one resequenced genome (supplementary table S3, Supplementary Material online). *Hme CTD10* and *22* were duplicated in two genomes (supplementary table S3, Supplementary Material online). *Hme CTD15, 20, 24* and *25* were duplicated in three genomes (supplementary table S3, Supplementary Material online). *Hme CTD19* was duplicated in four genomes (supplementary table S3, Supplementary Material online). *Hme CTD16* was duplicated in 5 genomes and *17*-*18* were duplicated in 7 of the 18 resequenced genomes (supplementary table S3, Supplementary Material online). Multiple CRAL-TRIO domain genes were also deleted in at least one resequenced genome: *Hme CTD11*, *15-23*, *25*, *27-28*, *30* and *32* (fig. 2*A*). Of these, *Hme CTD11*, *15*, *21* and *30* were deleted in one resequenced genome (fig. 2*B* andsupplementary table S4, Supplementary Material online). *Hme CTD23*, *28*, and *32* were deleted in two genomes (fig. 2*B* andsupplementary table S4, Supplementary Material online). *Hme CTD22* and *27* were deleted in three genomes (fig. 2*B* andsupplementary table S4, Supplementary Material online). *Hme CTD18-20* were deleted in four genomes (fig. 2*B* andsupplementary table S4, Supplementary Material online). *Hme CTD16-17* and *25* were deleted in five genomes (fig. 2*B* andsupplementary table S4, Supplementary Material online). One sequence identified in the de novo transcriptome, *Hme CTD26*, was excluded from analysis because the translation of the mRNA contig included stop codons and BLAST results suggested it was a chimeric sequence of *Hme CTD24* and *25*, most likely due to a Trinity misassembly. In some instances, duplications and deletions are large enough to change the presence or absence of a few genes in close proximity. Genes with the most duplications/deletions were duplicated/deleted in different subspecies; this shows that there is CNV between and within subspecies.

Seven genes (*Hme CTD34-40*) were found on chromosome 4; none were duplicated but *CTD36* was deleted in one *H. m. amaryllis* and one *H. m. aglaope*, and *CTD38*-*39* were both deleted in one *H. m. melpomene* genome (fig. 2*B* andsupplementary table S4, Supplementary Material online). One CRAL-TRIO domain gene (*Hme CTD41*) was located on chromosome 12, this gene was duplicated in one *H. m. aglaope* genome (fig. 2*B* andsupplementary table S3, Supplementary Material online). Lastly, *Hme CTD42*-*44* were on chromosome 21; *CTD41* was duplicated in one genome, *CTD42* was duplicated in one genome and deleted in four genomes, *CTD43* was duplicated in three and deleted in two genomes, and all resequenced genomes had one copy of *CTD44* (fig. 2*B* andsupplementary tables S3 and S4, Supplementary Material online). To summarize, we found potential CNV in 32 of the 43 CRAL-TRIO domain genes. Intriguingly, we found no CNV in *Hme CTD31*, our candidate chromophore-binding protein (see below).

We refer to our findings of structural variation as “potential” duplications or deletions because the results were derived through bioinformatic inference which is subject to error (Emerson et al. 2008; Alkan et al. 2011). Pindel uses read mapping information in order to find paired reads in which one read maps to the reference and the other mate does not to identify break points and direction of unmapped reads (Ye et al. 2009). For a few large areas with a lot of potential structural variation, Pindel could not differentiate whether the break was due to a duplication or deletion. Although current CNV analyses are subject to error, finding replication of duplications or deletions in more than one resequenced genome as we found in some instances is evidence that these results are meaningful. We investigated the breakpoints for genes that were duplicated/deleted in multiple resequenced genomes and found that a majority of genes had similar breakpoints in at least two individuals (supplementary tables S3 and S4, Supplementary Material online). In addition, a different study investigated CNVs in *H. melpomene rosina* using three discovery methods and found support for duplications in the genome location of *Hme CTD5-9* and *CTD16-18* (Pinharanda et al. 2016, 2017). That study also used Pacific Biosciences (PacBio) long molecule sequencing of *H. melpomene* and *H. cydno* to validate the findings of CNVs on chromosome 2. They found support for CNV in *Hme CTD10-12* using one discovery method and found many instances of CNVs in scaffold Hmel202006 using the three discovery methods (Pinharanda et al. 2016, 2017).

Twenty of our CRAL-TRIO domain genes were located on scaffold Hmel202006 including the genes within the *H. melpomene* expansion (*Hme CTD16-20* and *24-25*). We find the most CRAL-TRIO domain genes in tandem at a scaffold where our study and another found a large amount of CNV (Pinharanda et al. 2016, 2017). An interesting observation of CNV in this gene family was that all of the genes within the *H. melpomene* CRAL-TRIO expansion have potential CNV between individuals. In particular *Hme CTD16-20* have potential CNV in the highest number of resequenced genomes (*n* = 9, 9, 8, 6, 5) relative to other CRAL-TRIO domain genes. These results suggest that this area in the genome could be a hotspot for structural variation potentially due to unequal crossing over because similar duplicates are located in tandem.

The adaptive significance of CNV is still under investigation. As mentioned previously, the number of chemosensory receptor genes present between and within animal species is variable (Nozawa and Nei 2008) and their distribution suggests CNV is the result of genomic drift that can lead to adaptive evolution (Nozawa and Nei 2008). In *Drosophila melanogaster*, duplications with functional sequences were found to be possibly beneficial (Dopman and Hartl 2007). CNV affects phenotypes through its direct influence on gene expression. In humans, CNV can lead to Mendelian and complex diseases by affecting gene dosage (Redon et al. 2006). The HapMap project found a substantial amount of CNV between humans, and an association analysis determined that most significant CNV-associations had a positive correlation between gene copy number and gene expression levels (Stranger et al. 2007). Several positively selected duplication and deletion events in *D. melanogaster* have also been linked to gene expression variation (Emerson et al. 2008; Schmidt et al. 2010; Catalán et al. 2016).

Studies in *Drosophila* suggest CNV persists due to positive selection on paralogs that have tissue-specific expression (Dopman and Hartl 2007). To determine expression patterns for CRAL-TRIO domain genes we looked at gene presence and absence in the head, antennae, legs, and mouth parts of male and female *H. melpomene* (*n* = 4/sex). Here, we refer to complete presence as having >1 TPM for all replicates, partial >1 TPM for at least one replicate but not all four, and absence as mRNA expression <1 TPM for all replicates (fig. 2*C*). Some genes varied in presence patterns between tissue types such as *Hme CTD22*, *28*, and *38* (fig. 2*C*). *Hme CTD4-9*, *12*-*15*, *20-21*, *23-25*, *30-35*, *39*, *41*, and *42* had different presence patterns between sexes for one or more of the tissues examined. Although patterns of gene presence or absence (fig. 2*C*) provide an idea of which genes are expressed and where, absolute and differential expression needs to be analyzed to detect potential gene functions (see below).

CNV may be one contributor to the speciation of *Heliconius*, which has undergone a radiation in Central and South America (Kozak et al. 2015; Pinharanda et al. 2017). A recent study sought to identify adaptive CNV between two sympatric hybridizing species with distinct wing patterns, *H. melpomene* and *H. cydno* (Pinharanda et al. 2017). That study found four duplications with strong signals of divergent selection: these included an odorant binding protein, a serine protease, a regulator of the cell cycle and nitrogen compound metabolic processes, and one near the gene *cortex* which regulates wing color patterns (Nadeau et al. 2016; Pinharanda et al. 2017). The identification of an odorant binding protein supports the finding of *Heliconius* species having CNV of olfactory and gustatory receptor genes for putative host plant recognition in oviposition behavior (Briscoe et al. 2013). Divergent selection of a serine protease could be associated with *Heliconius* pollen feeding behavior (Smith et al. 2016). This raises the question as to what is the function of the CRAL-TRIO domain genes which have potential CNV between and within species.

### Differential Expression Analysis

Members of the CRAL-TRIO domain protein family are believed to be involved in transporting hydrophobic molecules. In particular, a member of this gene family (*pinta*) transports the chromophore necessary for phototransduction in *Drosophila*, however we did not find an ortholog in Lepidoptera (fig. 1). To detect whether any of the CRAL-TRIO domain genes in *H. melpomene* might have this function, we did a differential expression analysis to identify CRAL-TRIO domain genes upregulated in head tissues (relative to antennae, legs, and mouth parts), potentially involved in vision. We built a reference transcriptome assembly consisting of 68,388 transcripts and 31,193 contigs with an N50 of 2,627. On an average ten million reads mapped to the transcriptome and each library averaged 79% read mapping (supplementary table S5, Supplementary Material online). The transcriptome was deposited in Dryad under data identifier doi:10.5061/dryad.857n9 and the raw RNA-Seq reads were deposited in ArrayExpress archive under accession E-MTAB-6249 and E-MTAB-6342.

Differential gene expression analysis comparing heads versus antennae yielded 4,868 Differentially Expressed (DE) contigs using qvalue and 1,173 using Bonferroni for false discovery rate correction (supplementary table S6, Supplementary Material online), 561 of these 1,173 contigs were upregulated in heads (table 1). Analysis of head versus legs mRNA gave 6,108 DE contigs using qvalue and 1,472 using Bonferroni (supplementary table S7, Supplementary Material online), of these contigs 928 were upregulated in heads. Heads versus mouth parts comparison yielded 6,176 DE contigs using qvalue and 1,486 using Bonferroni (supplementary table S8, Supplementary Material online), 914 of these were upregulated in heads (table 1).

**Table 1:** Summary of Differentially Expressed (DE) and Upregulated Contigs

	qvalue	Bonferroni	**Upregulated in Heads**^a^
Head vs. antennae	4,868	1,173	561
Head vs. legs	6,108	1,472	928
Head vs. mouth	6,176	1,486	914

a These contigs are upregulated in heads using a Bonferroni FDR correction.

### CRAL-TRIO Domain Genes Expression

**Fig. 3.**
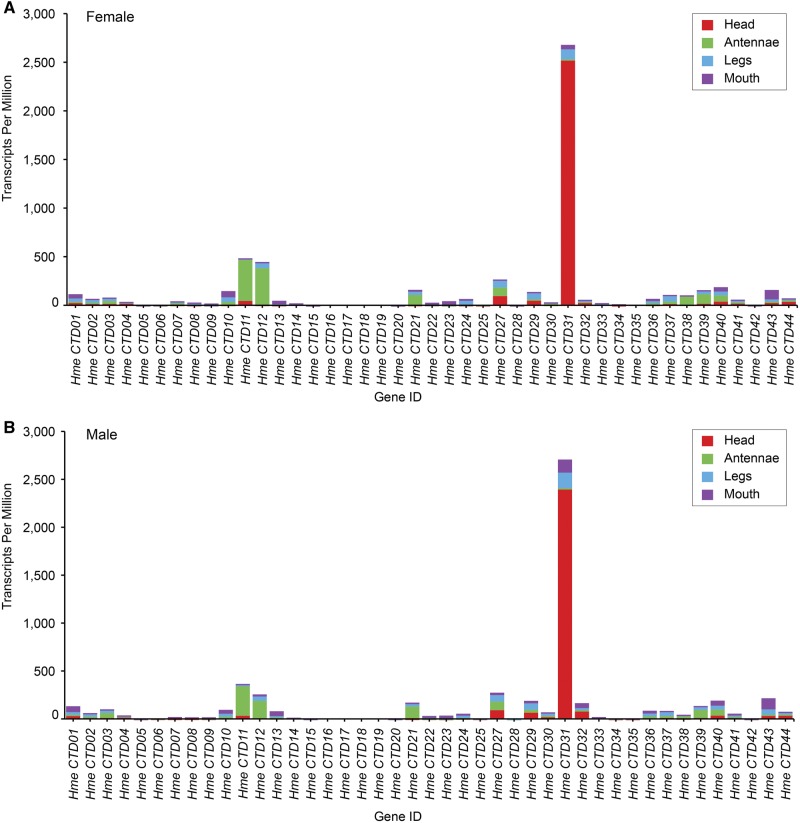
**—Expression of CRAL-TRIO domain genes.** (*A*) Transcripts per kilobase million (TPM) of CRAL-TRIO domain genes in female antennae, head, legs, and mouth parts. (*B*) TPM of CRAL-TRIO domain genes in male antennae, head, legs, and mouth parts.

To find if any CRAL-TRIO domain genes were potentially upregulated in *H. melpomene* heads, we inspected our significantly DE gene list for CRAL-TRIO domain genes. By using the Bonferroni method to correct for multiple tests, only one CRAL-TRIO domain contig was upregulated in the head versus antennae comparisons, *Hme CTD31* (table 2). This gene was also upregulated across comparisons when qvalue was used to correct for multiple tests. *Hme CTD22* was upregulated in head versus antennae and head versus legs when using qvalue, but *Hme CTD31* was the only contig upregulated across all comparisons. In addition, when we plotted the TPM for all genes across tissues, it became apparent that *Hme CTD31* is very highly expressed in male and female heads (fig. 3 and supplementary figs. S1–S4, Supplementary Material online).

**Table 2:** Head Expression Patterns of CRAL-TRIO Domain Contigs

	Qvalue			Bonferroni		
Gene ID	H vs. A	H vs. L	H vs. M	H vs. A	H vs. L	H vs. M
*Hme CTD1*	Not DE	Not DE	Down	Not DE	Not DE	Not DE
*Hme CTD2*	Not DE	Down	Down	Not DE	Not DE	Not DE
*Hme CTD3*	Not DE	Not DE	Not DE	Not DE	Not DE	Not DE
*Hme CTD4*	Down	Not DE	Not DE	Not DE	Not DE	Not DE
*Hme CTD6*	Not DE	Not DE	Not DE	Not DE	Not DE	Not DE
*Hme CTD8*	Not DE	Not DE	Not DE	Not DE	Not DE	Not DE
*Hme CTD9*	Down	Down	Down	Not DE	Not DE	Not DE
*Hme CTD10*	Down	Down	Down	Down	Down	Not DE
*Hme CTD11*	Down	Not DE	Not DE	Down	Not DE	Not DE
*Hme CTD12*	Down	Down	Down	Down	Down	Not DE
*Hme CTD13*	Down	Down	Down	Down	Not DE	Down
*Hme CTD14*	Down	Not DE	Not DE	Not DE	Not DE	Not DE
*Hme CTD15*	Not DE	Not DE	Not DE	Not DE	Not DE	Not DE
*Hme CTD21*	Down	Down	Down	Down	Down	Down
*Hme CTD22*	**Up**	**Up**	Down	Not DE	Not DE	Not DE
*Hme CTD29*	Not DE	Not DE	Not DE	Not DE	Not DE	Not DE
*Hme CTD30*	Down	Down	Not DE	Not DE	Not DE	Not DE
*Hme CTD31*	**Up**	**Up**	**Up**	**Up**	Not DE	Not DE
*Hme CTD32*	Not DE	Not DE	Not DE	Not DE	Not DE	Not DE
*Hme CTD34*	Down	Down	Down	Down	Down	Not DE
*Hme CTD35*	Down	Not DE	Not DE	Down	Not DE	Not DE
*Hme CTD36*	Down	Down	Down	Down	Down	Down
*Hme CTD37*	Down	Down	Down	Down	Down	Not DE
*Hme CTD38*	Down	Not DE	Not DE	Down	Not DE	Not DE
*Hme CTD39*	Down	Down	Not DE	Not DE	Not DE	Not DE
*Hme CTD40*	Down	Not DE	Down	Not DE	Not DE	Not DE
*Hme CTD41*	Down	Down	Down	Not DE	Not DE	Not DE
*Hme CTD43*	Not DE	Not DE	Down	Not DE	Not DE	Down

Not DE, not differentially expressed; Up, upregulated in heads; Down, downregulated in heads.

To investigate patterns of gene expression in the rest of the CRAL-TRIO domain genes, we used two-way ANOVAs to test if these genes varied by sex, tissue type, or sex and tissue type interaction (supplementary table S9, Supplementary Material online). We found that most genes varied by tissue type, including *Hme CTD1*, *2*, *4*, *5*, *7-14*, *21-24*, *27, 29*, *31*, *33-40*, *43*, and *44* (supplementary table S9 and figs. S5–S7, Supplementary Material online). Only two genes varied by sex *Hme CTD7* and *8* (supplementary table S9 and figs. S5–S7, Supplementary Material online).

The ANOVA analysis and the genome-wide DE analysis showed that *Hme CTD31* is a candidate pigment binding protein due to high expression in *H. melpomene* heads. The top NCBI blastp (protein to protein alignment) results for this gene are CRAL-TRIO domain containing protein and alpha-tocopherol transport protein. We found CRAL-TRIO domain genes that were upregulated in other tissues such as *Hme CTD11* and *CTD12* in the antennae. We do not know the specific function of these genes, but it is possible that they play a role in mediating the activation of other sensory receptors. Studies identifying chemosensory proteins have found some potential sensory receptors that are similar in sequence to opsins (Troemel et al. 1995). Opsins and some chemosensory receptors, such as olfactory, gustatory, and ionotropic receptors, belong to the rhodopsin-type superfamily of receptors but the groups vary in rate of molecular evolution. Opsins are more conserved between species, although gene duplications exist (see Sison-Mangus et al. 2008; Pohl et al. 2009; McCulloch et al. 2017), whereas olfactory, gustatory, and ionotropic receptors have duplicated extensively resulting in large gene families with a lot of copy number variation (Raible et al. 2006; van Schooten et al. 2016). Since these receptors have similar mechanisms of activation and similar functions in sensory perception, it is possible that the hydrophobic molecules with which they interact can be transported by proteins that are also similar to each other. In the cotton bollworm *H. armigera* four chemosensory proteins are expressed in both the eyes and proboscis; these proteins bind β-carotene and retinol (Zhu et al. 2016). That study demonstrates that proteins belonging to a family that responds to chemicals can have modified functions to have a role as a carrier for dietary carotenoids and visual processing in insects. Likewise, it is possible that *Hme CTD11* and *12*, upregulated in antennae, have functions in mediating olfaction through subfunctionalization.

### Hme CTD31 Candidate Chromophore Transporter

**Fig. 4.**
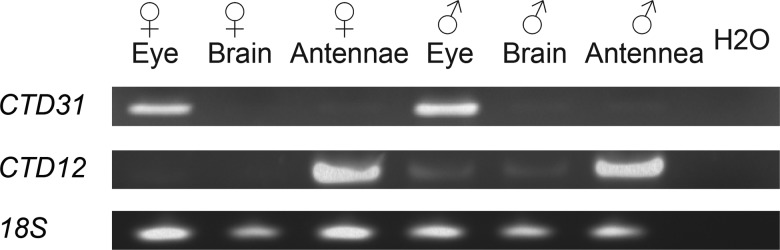
**—*Hme CTD31* RT-PCR.** RT-PCR of *Hme CTD31*, *Hme CTD12*, and *18 S* in female and male eye, brain, and antennae.

*Hme CTD31* is a candidate gene to explore for functions in visual pigment transport due to its upregulation in heads. However, head libraries were generated using whole head mRNA, so we used reverse transcription PCR (RT-PCR) to dissect whether *Hme CTD31* was expressed in the eye, brain, or both. We used the 18 S rRNA gene as a positive control for normalized mRNA presence. We also used *Hme CTD12* and antennae tissue to validate TPM expression patterns. We expected to see *Hme CTD31* expressed in the eye and brain but not in the antennae, and *Hme CTD12* only expressed in the antennae. *Hme CTD12* was only amplified in the antennae as expected (fig. 4). However, RT-PCR showed that *Hme CTD31* was only expressed in male and female eyes and not in the brain or the antennae (fig. 4). Additional support for *Hme CTD31* having a potential role in butterfly vision came from exploring the expression of CRAL-TRIO domain genes in heads of a different butterfly species, *B. anynana* (Macias-Muñoz et al. 2016) (accession numbers E-MTAB-3887 and doi: 10.5061/dryad.f98s6). We found that the *B. anynana* ortholog of *Hme CTD31* is the most highly expressed CRAL-TRIO domain gene in *Bicyclus* butterfly heads (supplementary fig. S8, Supplementary Material online) further supporting that expression of this gene is important in the compound eye across butterfly species.

**Fig. 5.**
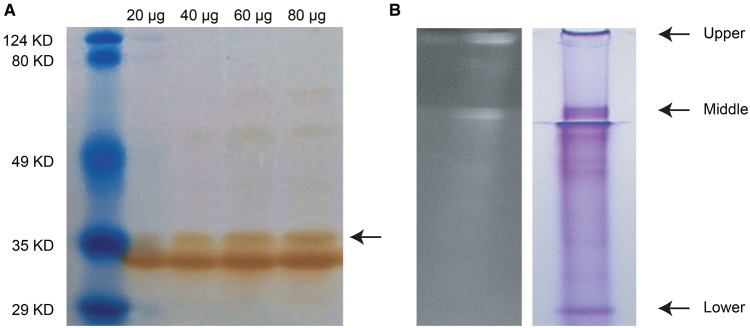
**—*Hme CTD31* Western Blot.** (*A*) Western blot using head tissue and Hme CTD31 antibody performed by Zyagen (San Diego CA). Arrow indicates expected band. (*B*) Butterfly head protein run on a native gel shows three bands that fluoresce under UV light. Arrows indicate the location of the upper, middle, and lower bands which were cut out and sequenced using mass spectrometry.

To localize where the Hme CTD31 protein is expressed in the *H. melpomene* eye, we designed an antibody against a unique peptide to perform immunohistochemistry. Our protein of interest has a predicted weight of ∼35 kDa, and an immunoblot of proteins extracted from whole head tissue using this antibody indicates it binds to a protein of the expected size (fig. 5*A*). We saw another band <35 kDa and that maybe the same protein but running through the gel differently due to phosphorylation of specific residues in the protein. Hme CTD31 has sites that are potentially phosphorylated with a probability score <0.75 at sites 7, 74, 109, 127, 175, 233, 275, and 28 (Blom et al. 1999).

**Fig. 6.**
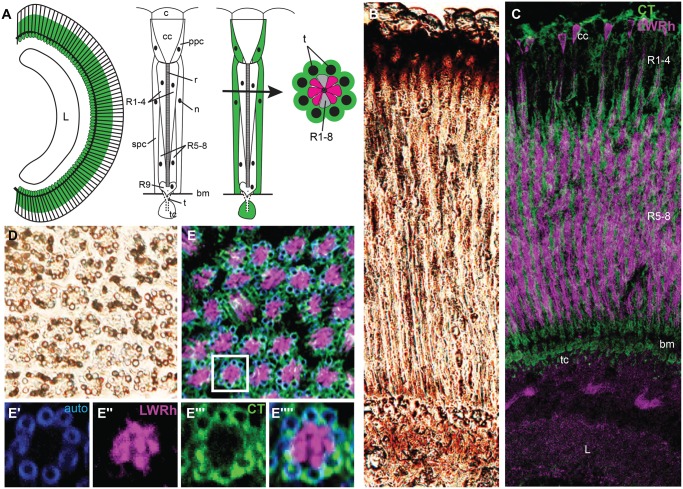
**—Immunohistochemistry of Hme CTD31 in *Heliconius melpomene* eye and optic lobe. (** *A*) Drawing of a longitudinal view of a compound eye and lamina, and longitudinal and transverse sections of a single ommatidium. Green highlights where we find Hme CTD31 expression; L, lamina; c, cornea; cc, crystalline cone; ppc, primary pigment cells; r, rhabdom; R1-9 conventional Lepidoptera numbering of photoreceptor cells; n, cell nucleus; spc, secondary pigment cells; bm, basement membrane; t, trachea; and tc, tracheal cell. (*B*) Brightfield longitudinal section showing pigments in the *H. melpomene* retina. (*C*) Longitudinal section with Hme CTD31 and LW opsin staining; Hme CTD31 is in green and LW opsin is in magenta. (*D*) Brightfield image of a transverse section of a butterfly eye, pigment is seen in the structures surrounding the ommatidia. (*E*) Transverse view of a butterfly eye stained for LW and Hme CTD31. (*E*′) autofluorescence showing tracheoles surrounding an individual ommatidium. (*E*′′) LW opsin staining showing where the LW photoreceptor cells are. (*E*′′′) CTD31 staining showing where the CRAL-TRIO domain protein Hme CTD31 is expressed. (*E*′′′′) merged image of LWRh, CTD31, and autofluorescence.

Next, to identify the cellular localization of the protein we examined longitudinal and transverse sections of the butterfly compound eye (fig. 6*A*). Each *Heliconius* ommatidium consists of a cornea, crystalline cone, and nine photoreceptor cells with a fused rhabdom and a tiered cell body arrangement. Primary pigment cells surround the crystalline cone and secondary pigment cells surround the photoreceptor cells. Brightfield images of a longitudinal section of the compound eye showed that there is pigment at the top of the ommatidia, around or within each ommatidium for its entire length, and below the basement membrane in tracheal cells (fig. 6*B*). A transverse image showed that the ommatidia are surrounded by eight tracheoles which have pigment along the tracheal walls (fig. 6*D*).

We used polyclonal antibodies against Hme CTD31 and the long wavelength opsin (LWRh) to visualize where Hme CTD31 was expressed in relation to photoreceptor cells (McCulloch et al. 2016). We found that Hme CTD31 is found in the primary pigment cells, secondary pigment cells, and tracheal cells (fig. 6*C*). The tracheal cells project tracheoles up and around the ommatidia, and these structures autofluoresce under blue light (488 nm laser) due to the presence of chitin (fig. 6*E*) (Iwata et al. 2014). Hme CTD31 is also expressed in the cell bodies surrounding the tracheole walls (fig. 6*E*). Hme CTD31 immunohistochemical results were similar to those of a retinol binding protein in the family Papilionidae, *Papilio* retinol binding protein (RBP). *Papilio* RBP binds retinol and was found to be expressed in primary pigment cells, secondary pigment cells, and tracheal cells (Wakakuwa et al. 2004). However, Hme CTD31 is expressed in the lower two-thirds of the ommatidia, rather than along the entire length, whereas *Papilio* RBP is found in the entire length of the ommatidia. The difference in where *Papilio* RBP and Hme CTD31 are located in *Papilio* and *Heliconius*, respectively, might be due to the difference in ommatidium morphology. *Papilio* RBP also does not belong to the CRAL-TRIO domain gene family. However, an ortholog of the gene encoding *Papilio* RBP in *H. melpomene* (Hme comp30064) was upregulated in heads relative to other tissue types (supplementary tables S6–S8 and fig. S9, Supplementary Material online). It is possible that Hme CTD31 and *Papilio* RBP are both necessary to transport the retinal molecule in different configurations as in vertebrates (McBee et al. 2001). The study characterizing *pinta* suggested there might be additional proteins in the primary pigment cells that are required for biosynthesis of the chromophore (Wang and Montell 2005).

It is also possible that Hme CTD31 functions in binding filtering pigments. From the RT-PCR and immunohistochemistry alone, we cannot confirm to what molecule Hme CTD31 binds but its upregulation in heads and localization in the ommatidia suggest that this protein has a role in butterfly vision. To confirm whether Hme CTD31 binds a chromophore, proteins from butterfly heads were run on a native gel and examined under UV light (fig. 5*B*). In the swallowtail butterfly, *Papilio* RBP bound to the chromophore fluoresces under UV light (Wakakuwa et al. 2004). We found three fluorescing bands which were cut and sequenced using mass spectrometry. Our candidate protein Hme CTD 31 is one of the top 20 proteins matching peptide fragment fluorescing upper (consisting of insoluble material), middle, and lower bands as detected by mass spectrometry (table 3). This evidence further supports our hypothesis that Hme CTD31, a CRAL-TRIO domain containing protein, is binding the chromophore molecule in butterflies. Hme CTD31 likely transports the vitamin-A derived chromophore molecule similar to vertebrate CRALBP and *Drosophila* PINTA. *Drosophila cralbp* and *pinta* both belong to the CRAL-TRIO and SEC14 superfamilies yet PINTA is the one shown experimentally to bind retinal (Wang and Montell 2005). Similarly, RALBP also belongs to the SEC14 superfamily and also functions in retinal binding in cephalopods (Ozaki et al. 1994; Speiser et al. 2014). These previous findings and our results suggest a conserved role for at least some of the CRAL-TRIO domain proteins, even if the specific function in this pathway is undertaken by nonorthologous members of the expanded gene family.

**Table 3:** Top 20 Proteins from Upper, Middle, and Lower Bands Detected by Mass Spectrometry Sorted by Upper Band Mascot Score

Accession	Protein Family	Upper		Middle		Lower	
		Mascot Score	Peptide Matches	Mascot Score	Peptide Matches	Mascot Score	Peptide Matches
comp33735_c0	Rfabg	5766	198	1699	67	45	2
comp31078_c1	Atpalpha	2204	62	1557	41	561	16
comp31397_c0	betaTub56D	1587	47	925	26	594	18
comp27767_c0	Vha68-2	1542	39	946	24	298	12
comp32095_c0	ATPsynbeta	1488	41	855	25	719	25
comp28890_c0	Gapdh2	1204	33	820	26	368	8
comp15204_c0	kdn	1187	31	787	24	660	17
comp27239_c0	CG1635	1051	29	692	18	127	2
comp31202_c0	alpha-Spec	998	38	1262	51	39	3
comp26414_c0	PyK	997	30	784	19	483	13
comp31948_c0	Pp2A-29B	963	20	475	17	584	19
**comp29636_c0**	**CG2663**	**947**	**43**	**514**	**24**	**494**	**24**
comp33018_c0	TER94	890	29	534	15	227	10
comp30615_c0	nrv3	867	19	786	21	139	4
comp14607_c0	Pgi	836	24	667	23	358	11
comp29963_c1	Gdh	833	29	1103	40	677	27
comp28746_c0	blw	822	27	588	19	57	3
comp25729_c0	Hsc70-4	817	32	2565	93	945	32
comp29025_c0	Mdh2	817	25	764	21	200	7
comp31520_c0	alphaTub84B	741	26	609	16	262	10
comp30064_c0	CG10476	517	12	271	5	309	7

Note.—Mascot protein scores >67 are significant (*P* < 0.05). Bold indicates comp29636_c0, which corresponds to CTD31. comp30064 corresponds to the *Papilio* RBP homolog in *Heliconius melpomene*.

To summarize, we investigated a large gene family whose function in insects is only known for one gene in one species: *pinta* transports the chromophore molecule in *Drosophila* and is necessary for phototransduction. Although other members of the CRAL-TRIO domain gene family have undergone an expansion, we found no ortholog of the *pinta* gene in Lepidoptera. In *H. melpomene*, we found an expansion of genes in close proximity suggesting that CRAL-TRIO domain genes are evolving by tandem duplications. We also found copy number variation of CRAL-TRIO domain genes between individuals. Although the function of these genes is not known, we hypothesized that one or more of these genes could have a role in vision similar to *pinta* and we were able to identify one candidate gene upregulated in *H. melpomene* heads and two other genes upregulated in antennae. This gene, *Hme CTD31*, was found in eye mRNA and its protein product was localized to secondary and primary pigment cells and to a protein gel band that fluoresces under UV light. Interestingly, *Hme CTD31* is a single copy gene across the 18 resequenced genomes we investigated, suggesting a critical function. We have thus identified a CRAL-TRIO domain containing gene that likely encodes a chromophore binding protein in butterflies, a paralogous member of the *pinta* gene family that is rapidly evolving in butterflies.

## Supplementary Material

Supplementary data are available at *Genome Biology and Evolution* online.

## Supplementary Material

Supplementary Figures and Tables
