# Supplementary material for: Copy Number Variation and Expression Analysis Reveals a Nonorthologous Pinta Gene Family Member Involved in Butterfly Vision
Source: Genome Biol Evol. 2017 Nov 9;9(12):3398–412. doi: 10.1093/gbe/evx230 (PMC5739039; doi:10.1093/gbe/evx230)
Supplement: Supplementary Figures and Tables [file evx230_supp.zip › Macias_GBE_supplementary_figs.pdf]

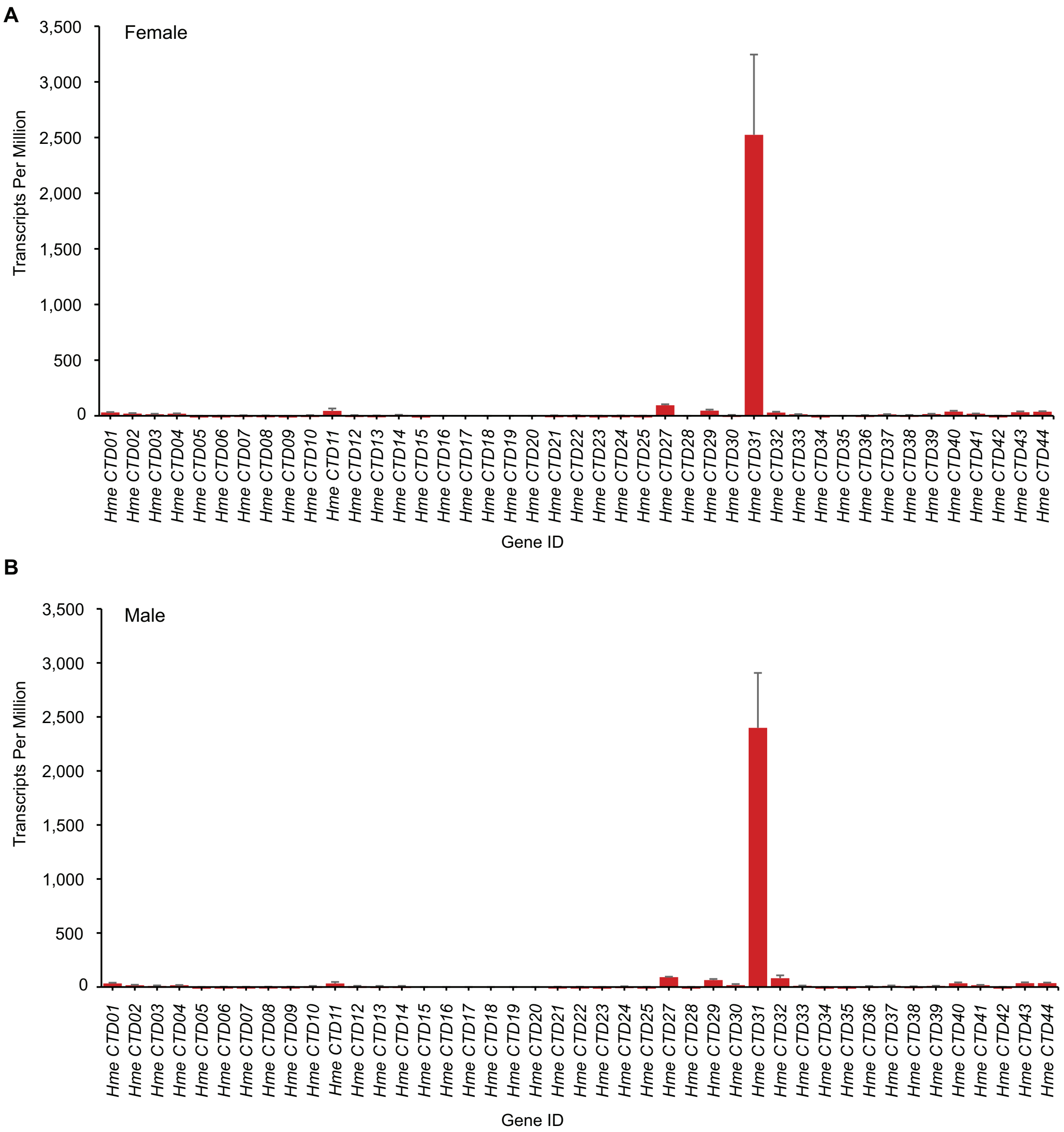

Fig S1. CRAL-TRIO domain gene expression in *H. melpomene* heads. (A) Transcripts Per Kilobase Million (TPM) of CRAL-TRIO domain genes in *H. melpomene* female head with standard error bars. (B) Transcripts Per Kilobase Million (TPM) of CRAL-TRIO domain genes in *H. melpomene* male head with standard error bars.

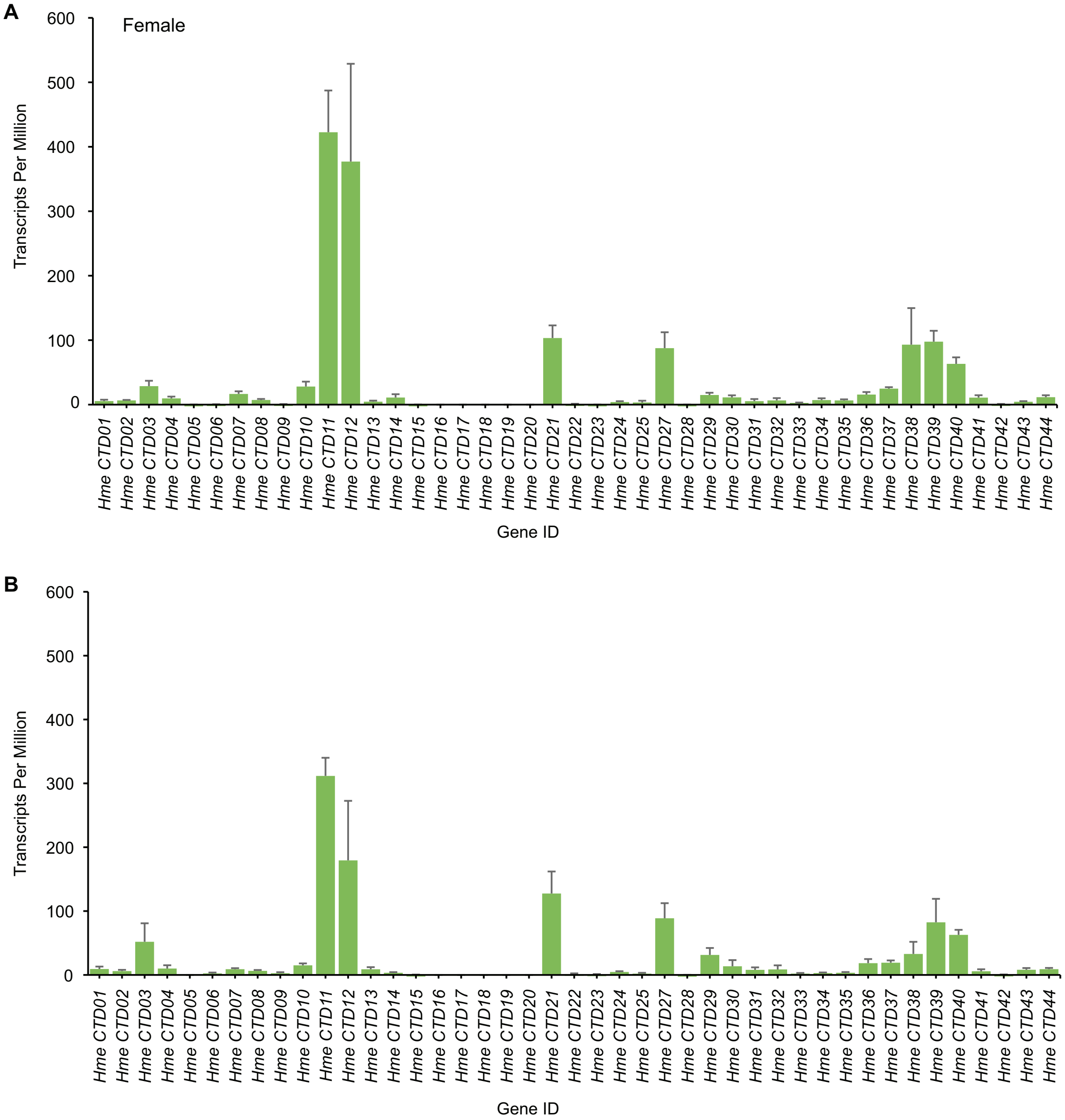

Fig S2. CRAL-TRIO domain gene expression in *H. melpomene* antennae. (A) Transcripts Per Kilobase Million (TPM) of CRAL-TRIO domain genes in *H. melpomene* female antennae with standard error bars. (B) Transcripts Per Kilobase Million (TPM) of CRAL-TRIO domain genes in *H. melpomene* male antennae with standard error bars.

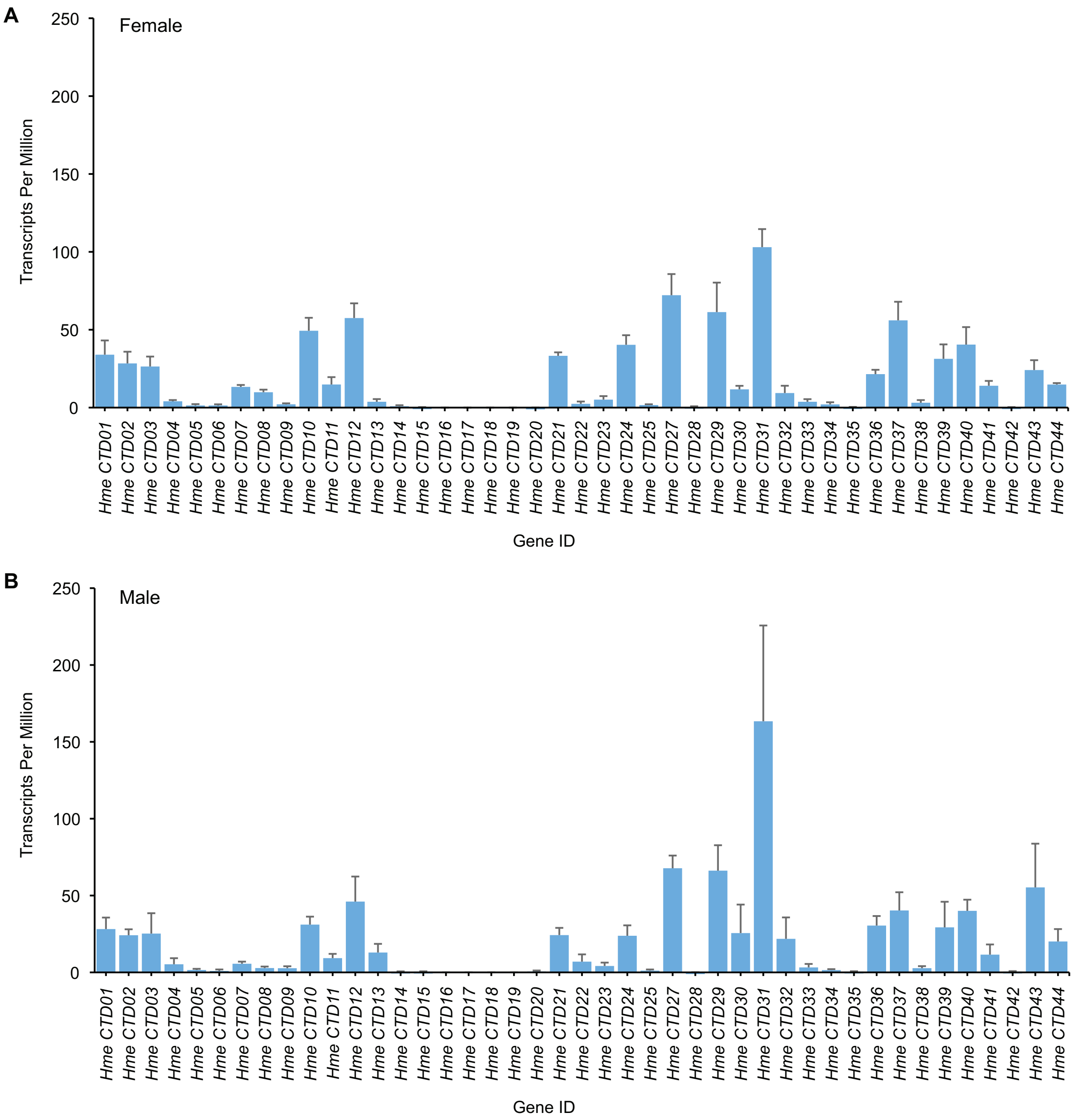

Fig S3. CRAL-TRIO domain gene expression in *H. melpomene* legs. (A) Transcripts Per Kilobase Million (TPM) of CRAL-TRIO domain genes in *H. melpomene* female legs with standard error bars. (B) Transcripts Per Kilobase Million (TPM) of CRAL-TRIO domain genes in *H. melpomene* male legs with standard error bars.

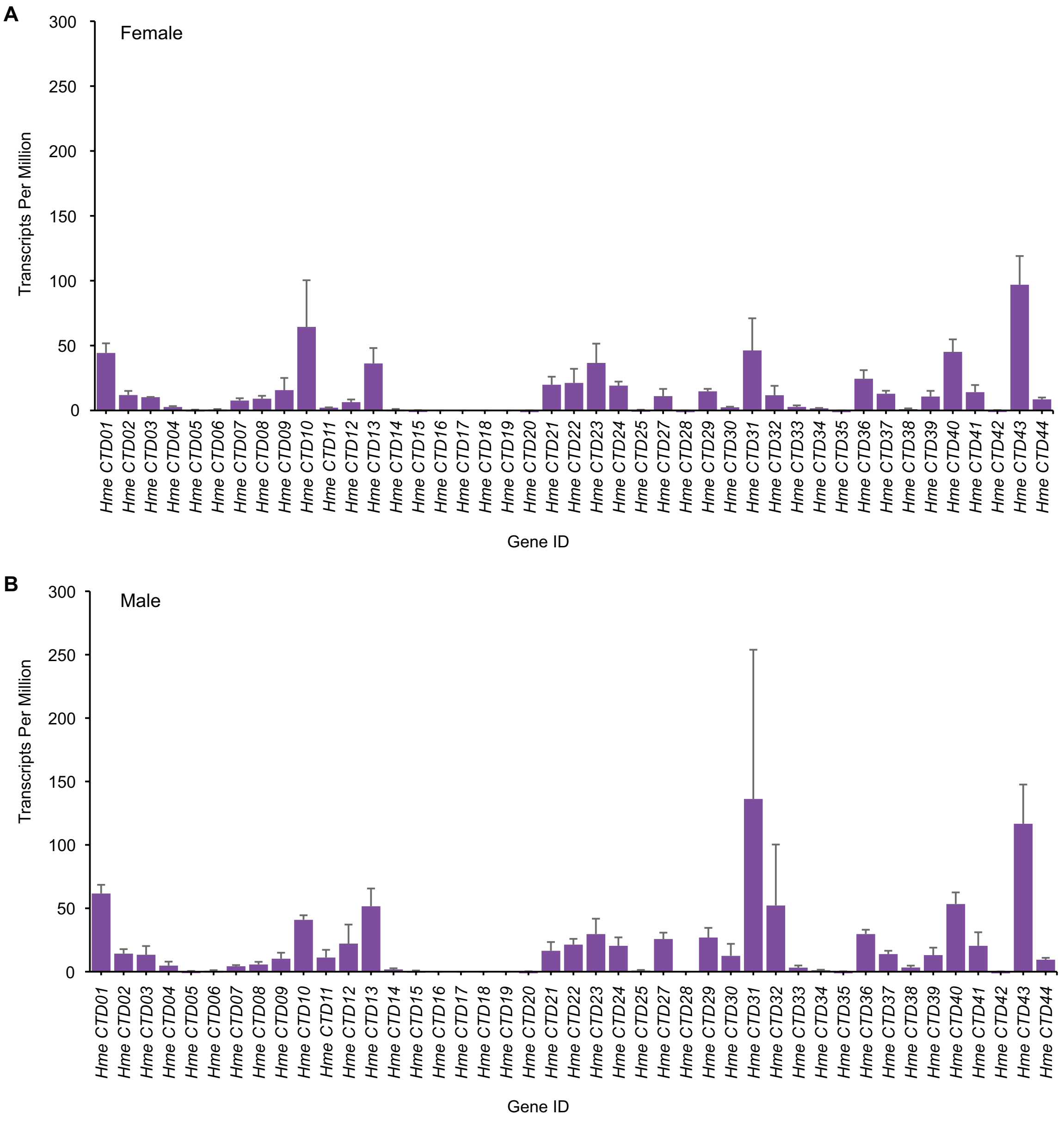

Fig S4. CRAL-TRIO domain gene expression in *H. melpomene* mouth. (A) Transcripts Per Kilobase Million (TPM) of CRAL-TRIO domain genes in *H. melpomene* female mouth parts with standard error bars. (B) Transcripts Per Kilobase Million (TPM) of CRAL-TRIO domain genes in *H. melpomene* male mouth parts with standard error bars.

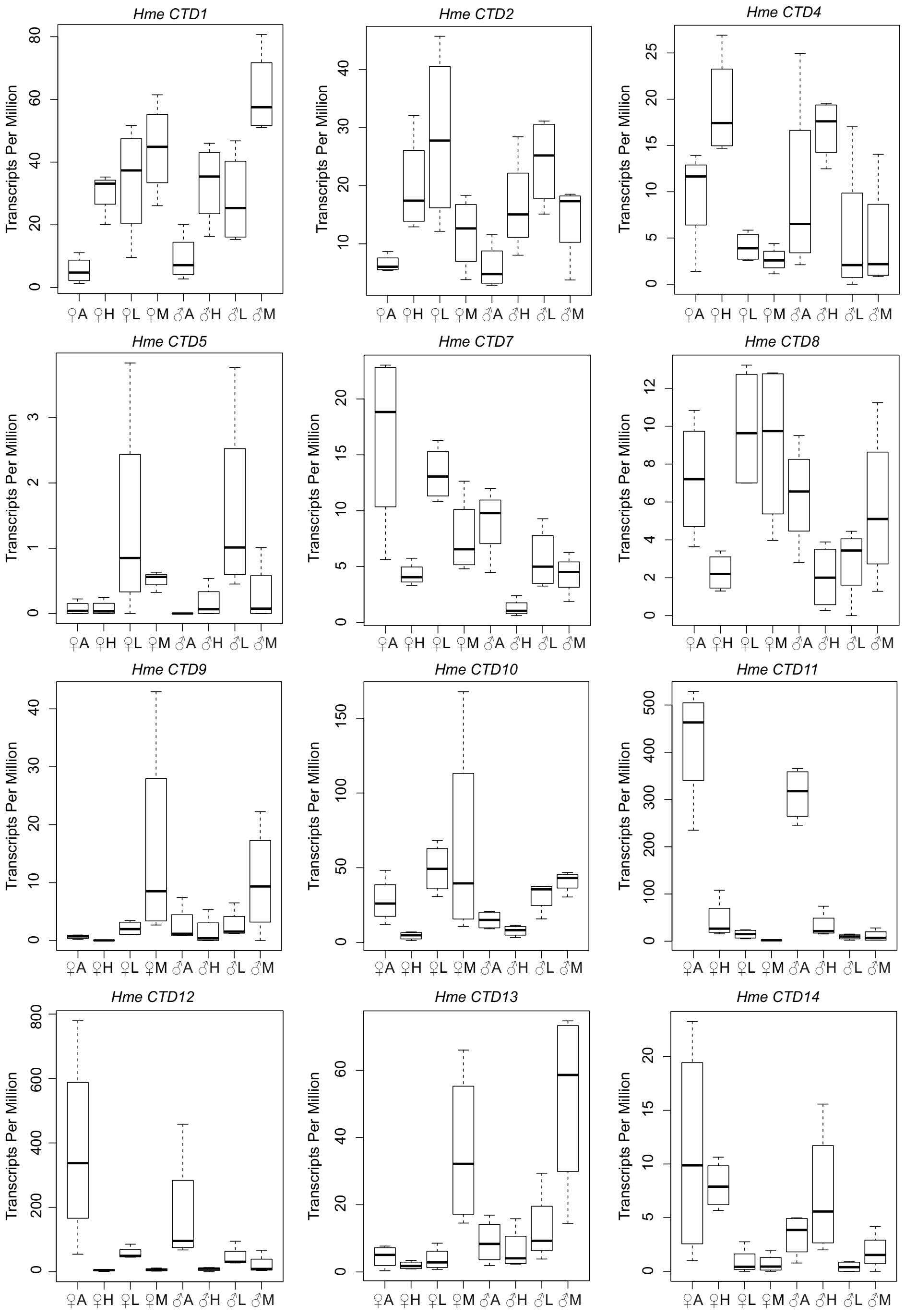

Fig S5. TPM plots of CRAL-TRIO domain genes (*Hme CTD1-20*) with p <0.05 using ANOVA.

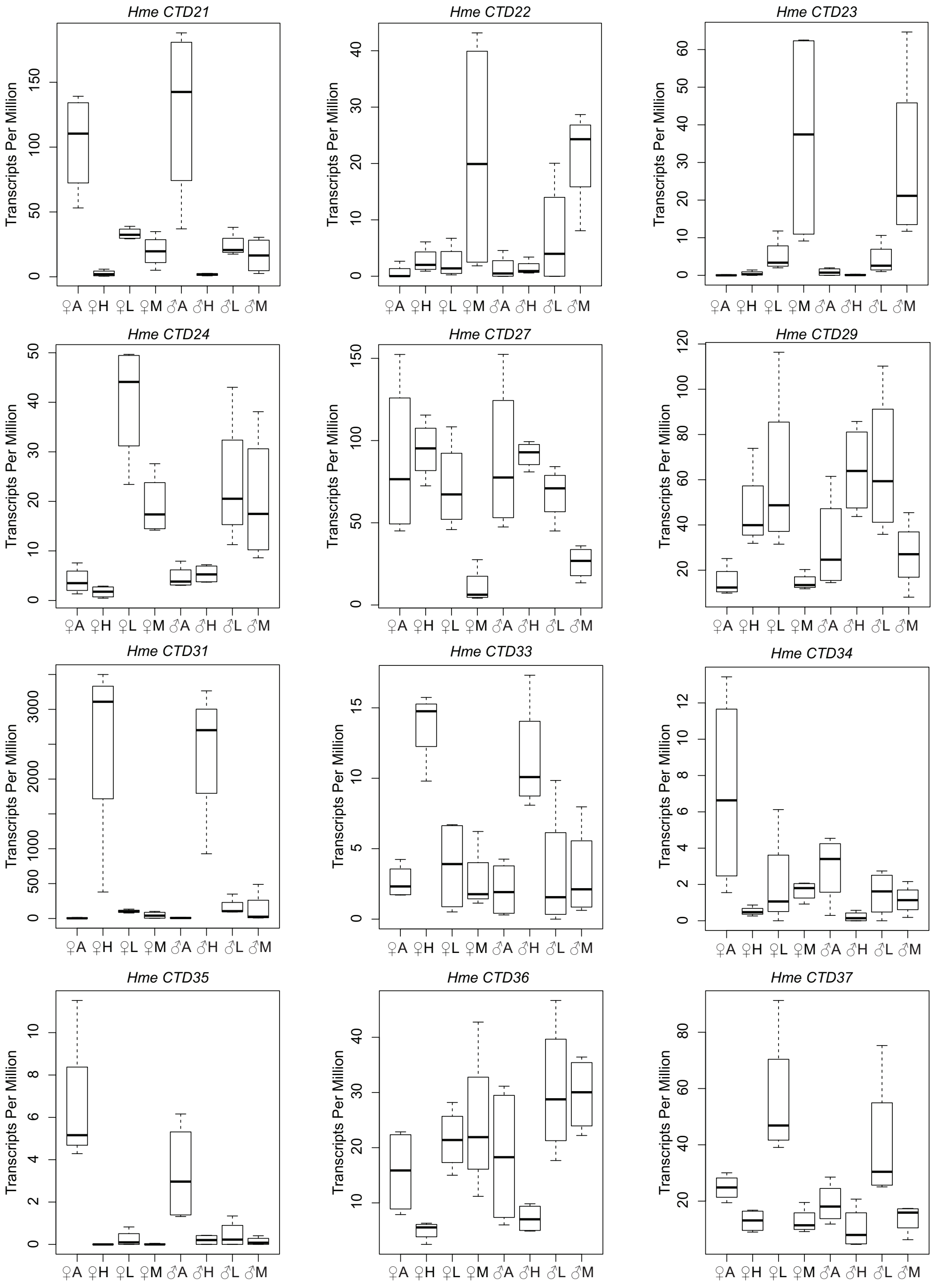

Fig S6. TPM plots of CRAL-TRIO domain genes (*Hme CTD21-37*) with p <0.05 using ANOVA.

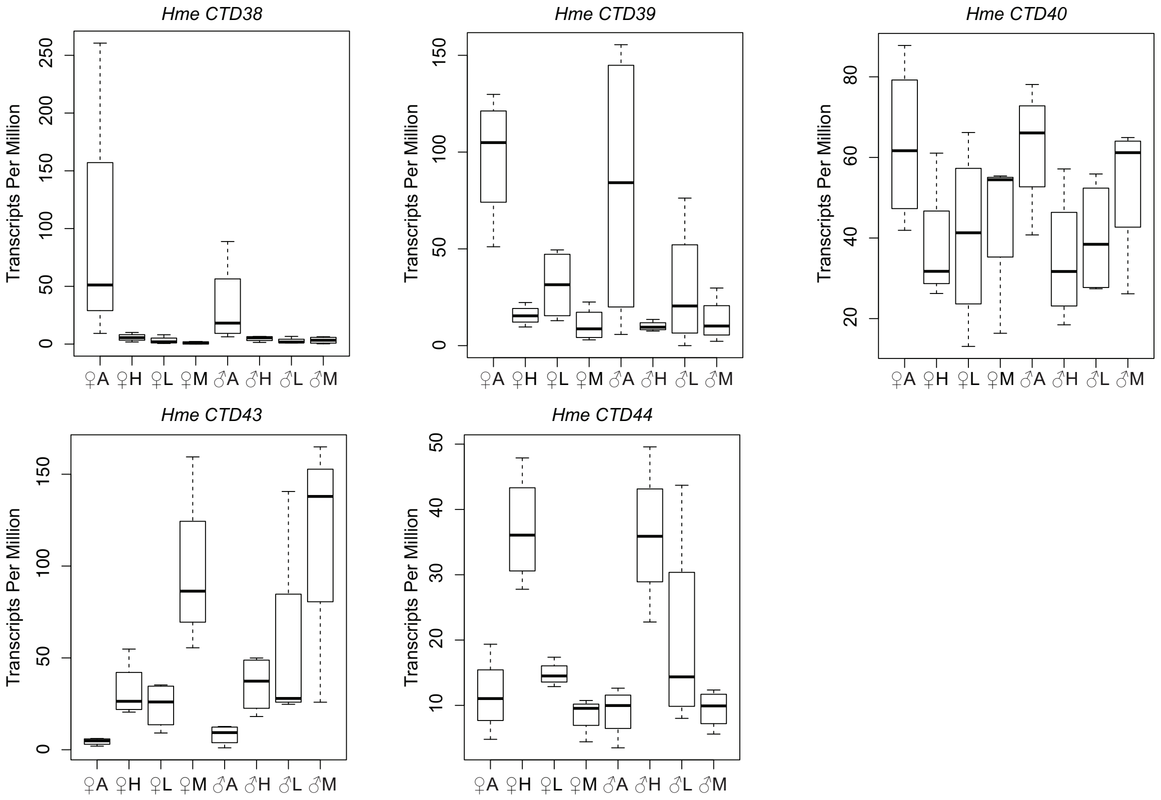

Fig S7. TPM plots of CRAL-TRIO domain genes (*Hme CTD38-44*) with p <0.05 using ANOVA.

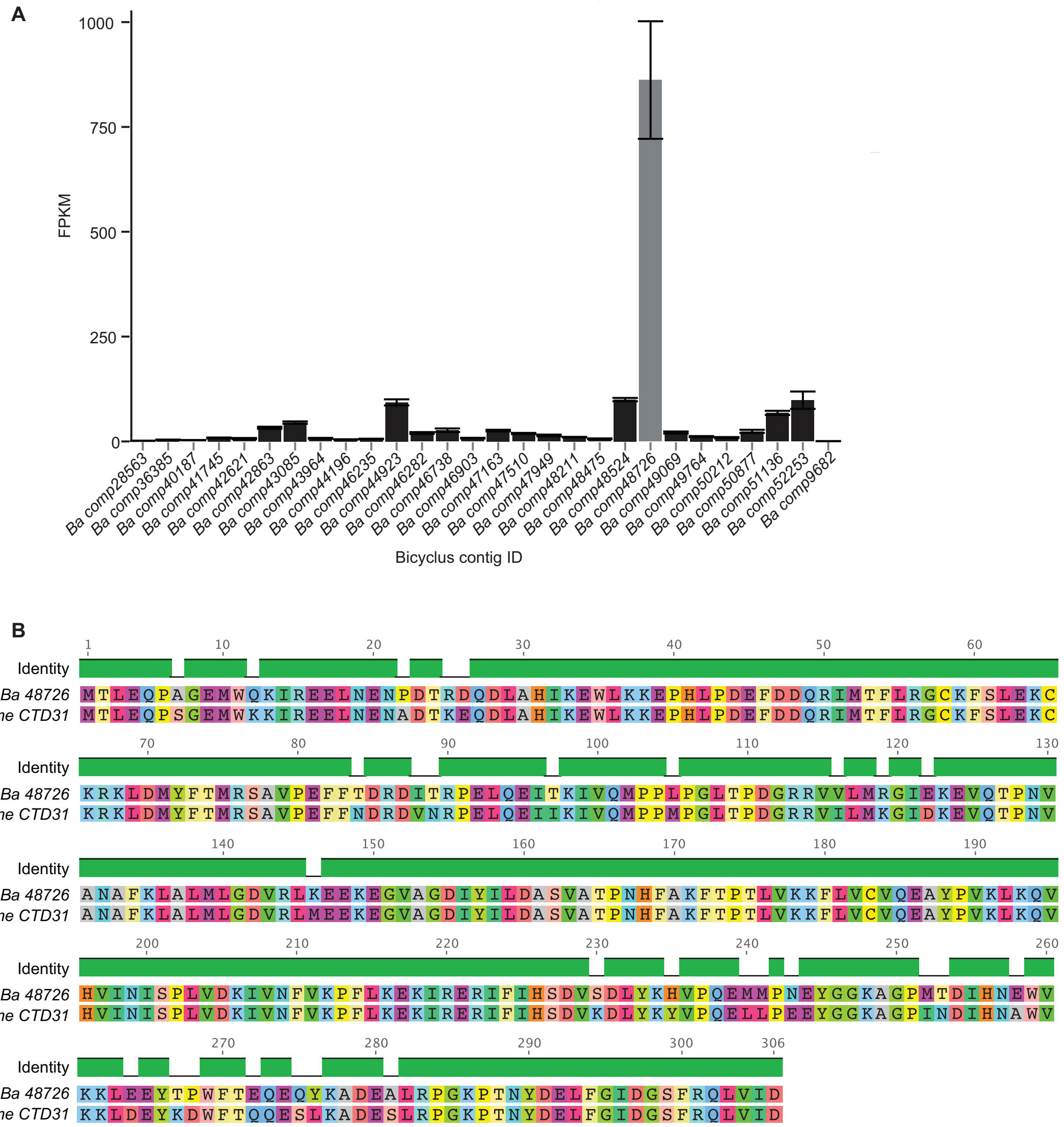

Fig S8. Expression of CRAL-TRIO domain genes in *Bicyclus anynana*. (A) FPKM plot of CRAL-TRIO domain genes in heads of the butterfly *B. anynana*. One gene, *BA comp48725* in grey, is highly expressed and the ortholog of *Hme CTD31*. (B) Alignment of *BA comp48725* and *Hme CTD31*.

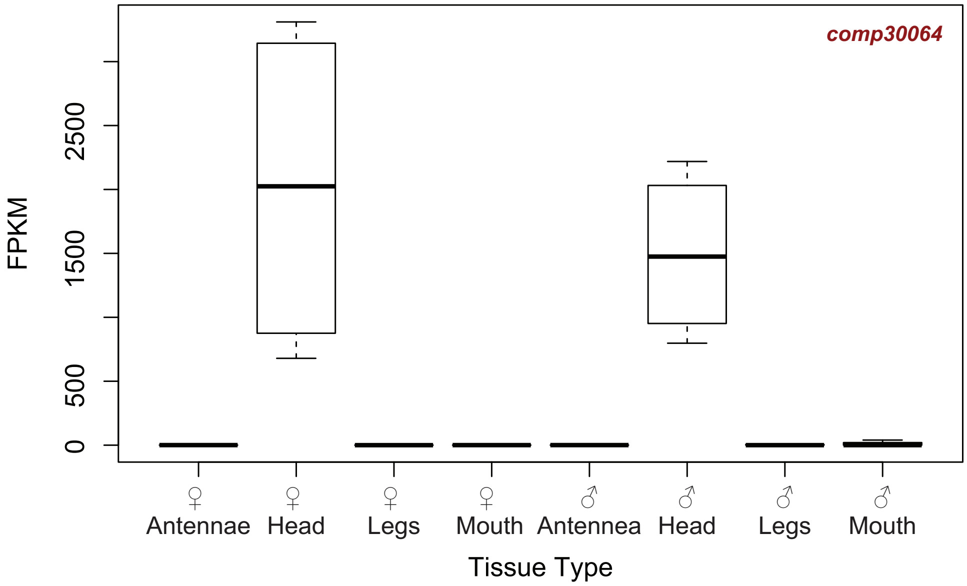

Fig S9. Expression of *Papilio RBP* in *H. melpomene* tissue types. FPKM plot of the *H. melpomene Papilio RBP* ortholog, *comp30064*. This gene encoding a retinol-binding protein is upregulated in *H. melpomene* male and female heads compared to other tissue types.
